# Supplementary material for: Optimization of Radiolabeling of a [90Y]Y-Anti-CD66-Antibody for Radioimmunotherapy before Allogeneic Hematopoietic Cell Transplantation
Source: Cancers (Basel). 2023 Jul 18;15(14):3660. doi: 10.3390/cancers15143660 (PMC10377894; doi:10.3390/cancers15143660)
Supplement: Supplementary file 1 [file cancers-15-03660-s001.zip › cancers-2488991-supplementary.docx]

# Supplementary

Optimization of radiolabeling of a [^90^Y]Y-anti-CD66-antibody for radioimmunotherapy before allogeneic hematopoietic cell transplantation

Gordon Winter^1, †,*^, Carmen Hamp^1,†^, Gabriel Fischer^1^, Peter Kletting^1^, Gerhard Glatting^1^, Christoph Solbach^1^, Hendrik Herrmann^1^, Elisa Sala^2^, Michaela Feuring^2^, Hartmut Döhner^2^, Ambros J. Beer^1^, Donald Bunjes^2^, Vikas Prasad^1,3^

^1^ Department of Nuclear Medicine, Ulm University Medical Center, 89081, Ulm, Germany; [gordon.winter@uni-ulm.de](mailto:gordon.winter@uni-ulm.de); [carmen.hamp@uniklinik-ulm.de](mailto:carmen.hamp@uniklinik-ulm.de); [gerhard.glatting@uniklinik-ulm.de](mailto:gerhard.glatting@uniklinik-ulm.de); [christoph.solbach@uniklinik-ulm.de](mailto:christoph.solbach@uniklinik-ulm.de); [hendrik.herrmann@uniklinik-ulm.de](mailto:hendrik.herrmann@uniklinik-ulm.de); [ambros.beer@uniklinik-ulm.de](mailto:ambros.beer@uniklinik-ulm.de)

^2^ Department of Internal Medicine III, Ulm University Medical Center, 89081, Ulm, Germany; [elisa.sala@uniklinik-ulm.de](mailto:elisa.sala@uniklinik-ulm.de); [michaela.feuring@uniklinik-ulm.de](mailto:michaela.feuring@uniklinik-ulm.de); [hartmut.doehner@uniklinik-ulm.de](mailto:hartmut.doehner@uniklinik-ulm.de); [donald.bunjes@uniklinik-ulm.de](mailto:donald.bunjes@uniklinik-ulm.de)

^3^ Mallinckrodt Institute of Radiology-Division of Nuclear Medicine, Washington University in St Louis, MO, USA; [drvikaspd@yahoo.com](mailto:drvikaspd@yahoo.com)

***** Correspondence: gordon.winter@uni-ulm.de

**Table S1.** Results of the manual integration of the radioactive HPLC peaks for serum stability test using [^90^Y]Y-DTPA-BN-CHX-A’’-anti-CD66-mAb for 0.5 h and 24 h after incubation start. The relative area under the peak is given in percent (%).

| Exp. | 30 min [%] | 24 h [%] |
| --- | --- | --- |
| 1 | 87.1 | 70.0 |
| 2 | 85.5 | 69.4 |
| 3 | 80.7 | 73.3 |
| 4 | 87.6 | 69.8 |
| Mean ± SD | 85.2 ± 3.2 | 70.7 ± 1.8 |

**Figure S1.** Chromatograms of the serum stability tests of [^90^Y]Y-DTPA-Bn-CHX-A''-anti-CD66-mAb. After injection of 20 µl sample, the main peak of the labeled mAb was detected at 8.5 min retention time, while the peak of free yttrium was detectable at 11.6 min (A). A small proportion of the antibody is in dimerized form and can be detected as a shoulder before the antibody signal (A, B). We assume that the dimerized portion is transient and can also fulfil the functions of the monomer. For this reason, the dimerized portion was included in the integration.
Due to the high concentration of radiolabeled mAb in a comparatively small volume, an increase in free yttrium and signals after the mAb main peak associated with defective antibodies can also be observed after 24h due to radiolysis (B). This effect is not expected due to a prompt application of the mAbs into the patient after processing, resulting in a lower concentration due to the significantly higher distribution volume.

**Figure S2.** Graphical representation of liver parameters of patient 3 previously treated with inotuzumab ozogamicin (A), and liver parameters of patient 4 who had baseline elevated transaminases (B). The time course of the absolute values of the parameters is plotted on the ordinate

**Figure S3.** Neutrophil counts of individual patients in giga/L (x10^9^ cells/L) were presented over time on days (D) after RIT (P1-P5; colored lines) in combination with the respective mean and standard deviation (black line). Neutrophil counts decreased to zero within 12 days after successful treatment.
